# Predictive value of systemic immune-inflammation index for pathological complete response in patients receiving neoadjuvant immunochemotherapy for locally advanced esophageal cancer

**DOI:** 10.3389/fsurg.2022.1091601

**Published:** 2023-01-04

**Authors:** Wu Han, Kai Weng, Peipei Zhang, Zhinuan Hong

**Affiliations:** ^1^ Department of Thoracic Surgery, Fujian Medical University Union Hospital, Fuzhou, China; ^2^ Key Laboratory of Cardio-Thoracic Surgery (Fujian Medical University), Fujian Province University, Fuzhou, China; ^3^ Key Laboratory of Ministry of Education for Gastrointestinal Cancer, Fujian Medical University, Fuzhou, China; ^4^ Fujian Key Laboratory of Tumor Microbiology, Fujian Medical University, Fuzhou, China

**Keywords:** esophageal squamous cell carcinoma, pathological complete response, systemic immune-inflammatory index, neoadjuvant immunochemotherapy, nomogram model

## Abstract

**Objectives:**

Neoadjuvant immunochemotherapy (nICT) has been confirmed with promising pathological complete response (pCR) among locally advanced esophageal squamous cell carcinoma (ESCC). However, there were still no reliable and accurate predictors to predict the treatment response. This study aimed to explore the predictive value of inflammatory and nutritional parameters.

**Methods:**

Patients with ESCC who underwent radical surgery after nICT between January 2020 and April 2022 were included in the study. First, the least absolute shrinkage and selection operator regression (LASSO) logistic regression analysis was used to screen independent inflammatory and nutritional parameters. Secondly, univariate and multivariate logistic regression were used to screen and predict independent risk factors for pCR. Thirdly, a nomogram was constructed based on the independent predictive factors, and 30% of the included population was randomly selected as the validation cohort. We used the receiver operating characteristic (ROC) curve, calibration curve, and decision curve analysis (DCA) curve to evaluate the nomogram model.

**Results:**

A total of 97 ESCC patients were screened for analysis, with 20 patients with pCR (20.32%). Only the systemic immune-inflammation index (SII) was screened after LASSO-logistic regression when *λ* was 0.06. The cut-off value of SII was 921.80 with an area under curve (AUC) value of 0.62. We defined SII > 921.80 as high SII and SII ≦ 921.80 as low SII. Further, the univariate and multivariate analysis further determined SII(OR = 3.94, 95%CI:1.26–12.42, *P* = 0.02) and clinical stage(OR = 0.35, 95%CI:0.12–0.98, *P* = 0.05) were independent predictive factors of pCR. One novel nomogram was established with an AUC value of 0.72 in the training cohort and 0.82 in the validation cohort. The Brier score of the calibration curve was 0.13. The calibration curve showed good agreement between the predicted results and the actual results in both the training cohort and the validation cohort. Compared with the clinical stage, the DCA confirmed a better clinical value of the nomogram model in both the training cohort and the validation cohort.

**Conclusions:**

High pretreatment SII and early clinical stage were independently associated with pCR among ESCC receiving nICT. We further established and validated one novel nomogram model to effectively predict pCR among ESCC after nICT.

## Introduction

Esophageal cancer is one of the most severe malignant tumors in the digestive system. Its incidence rate and mortality rate rank seventh and sixth among all malignant tumors in the world, respectively (1). China is one of the regions with the highest risk of esophageal cancer. More than 90% of esophageal cancer is squamous cell carcinoma, and the overall 5-year survival rate is less than 30% (2). For patients with locally advanced esophageal squamous cell carcinoma (LA-ESCC), the effect of simple surgical treatment is limited, and the incidence of postoperative recurrence and metastasis is high. Therefore, people put forward the concept of new adjuvant treatment to improve the survival rate of LA-ESCC patients (3).

In recent years, the application of immunotherapy has gradually matured, and many studies have confirmed the good therapeutic effect of neoadjuvant immunotherapy combined with chemotherapy (nICT) in LA-ESCC patients (4,5). Pathological complete remission (pCR) is one of the evaluation indicators of tumor neoadjuvant therapy, which can provide effective prognosis evaluation, postoperative follow-up, and individualized treatment guidance for patients. Preoperative CPS and TPS scores of PD-L1 could not effectively predict the degree of pathological reaction in patients receiving nICTin patients receiving nICT (6). The PALACE study indicated that the expression of PD-L1 wasn't obviously associated with the pathologic regression among patients receiving preoperative pembrolizumab combined with chemoradiotherapy (7). The NICE-2 study presented no significant correlation between PD-L1 expression and pathological response in ESCC patients receiving ocrelizumab and chemotherapy (8). Thus, it's of great significance to find simple and effective indicators to accurately predict the pathological response before treatment.

Previous studies have shown that inflammation plays a crucial role in the occurrence, development, and metastasis of tumors (9). Among many indicators reflecting host systemic inflammatory response, lymphocyte, neutrophil, and platelet counts in peripheral blood have been widely reported to be able to predict postoperative recurrence and long-term survival of patients with various malignant tumors and show a certain clinical application prospect (10–13). Recently, Feng J et al. showed that integrative inflammatory and nutritional score (IINS) before treatment was an independent predictor of pCR in patients with resectable LA-ESCC receiving nICT (14).

However, studies focused on predicting whether patients would achieve pCR were still limited. The purpose of this study was to explore the predictive value of inflammatory and nutritional parameters in the prediction of pCR among ESCC patients receiving nICT. Further, we also aimed to establish a novel nomogram model based on the independent predictive factor and hope to provide a reference for an individualized treatment plan.

## Methods

### Patient selection

This was a retrospective study based on prospectively collected data. This study was approved by the ethics committee of Fujian Medical University Union Hospital. The ethical approval number was 2022YK202. We conducted this analysis strictly adhering to the Declaration of Helsinki (1964). Consecutive patients who underwent nICT for ESCC after esophagectomy between January 2020 and April 2022 were identified.

Inclusion criteria included: pathological type was ESCC; cT3 + or cN + before treatment; ASA status ≤ III; without clinical signs of distant metastasis; undergoing radical esophagectomy. Exclusion criteria included: Patients who had unresectable tumors or metastases; Patients who received other induction therapy, such as neoadjuvant chemoradiotherapy or neoadjuvant immnochemoradiotherapy, or neoadjuvant chemotherapy.

### Treatment protocols

The treatment regimen received by patients in the NICT group was intravenous PD-1 inhibitors (pembrolizumab at a dose of 200 mg, sintilimab at a dose of 200 mg, toripalimab at a dose of 240 mg, tirelizumab at a dose of 240 mg, and camrelizumab at a dose of 200 mg) every three weeks (1 day) in combination with platinum-based chemotherapy and paclitaxel/docetaxel (CF / DF group). Previously we have completed two phase II clinical trials, and the details of neoadjuvant regimens were listed in previously published articles (15,16). For patients who completed two or three cycles of nICT, we clinically evaluated the patients again to determine whether the patients should undergo esophagectomy or continue the induction treatment. For patients suitable for radical esophagectomy, we conduct thoracoscopically assisted or robot-assisted McKeown minimally invasive esophagectomy (MIE) in 4–6 weeks after the last cycle of neoadjuvant therapy. We performed 2-field lymphadenectomy and used a 3.0–3.5 cm width tube stomach to replace the esophagus. When there were enlarged cervical lymph nodes, we conducted 3-field lymphadenectomy.

### Outcome measures

The primary outcome was pathological complete response (pCR), which was defined as no residual tumor in both the primary tumor and lymph nodes. Tumor regression grade (TRG) (modified Ryan scheme) 0 was equal to pCR (17). All specimens were systematically evaluated by an experienced pathologist, and if necessary pathological slides would be evaluated by another pathologist. The 8th AJCC/UICC TNM staging system was applied in this analysis.

The value of inflammatory and nutritional parameters was collected from the medical record system. The Neutrophils (NEU), platelet (PLT), lymphocyte(LY), monocyte (MONO), albumin (ALB), body weight, hemoglobin (HB), and body mass index(BMI) were obtained within one week before nICT. The PLR, NLR, and LMR were defined as PLTs divided by LYs, NEUTs divided by LYs, and LYs divided by MONOs respectively. The hemoglobin albumin lymphocyte platelet (HALP) was calculated as follows: HALP = HB × ALB × LY/PLT. The systemic immune-inflammation index (SII) was calculated as follows: SII = PLT × NEUT/LY. The systemic inflammation response index (SIRI) was calculated as follows: SIRI = MONO × NEUT/LY. The prognostic nutritional index (PNI) was calculated as follows: PNI = ALB (g/L) + 5 × LY (10^9^/L) (18).

### Statistical analysis

First, the patients were divided into pCR group and non pCR group. We use mean ± standard deviation or median (interquartile distance) to represent continuous data and use numbers (percentage) to represent classified data. Baseline characteristics and postoperative information were compared. Student t-test or Mann Whitney U test was used for continuous variables, and the chi-square test or Fisher exact test was used for categorical variables. Continuous variables were converted into categorical variables according to the best cut-off value of the ROC or clinical experience of the subjects. Secondly, considering there were a total of 18 inflammation and nutrition indicators with potential collinearity of variables, we used the least absolute shrinkage and selection operator regression (LASSO) regression model to screen variables. The principle of LASSO regression screening variables is to compress the regression coefficients of each variable in the form of penalty increment (12). In addition, we also cross-verified the Lasso regression model. Thirdly, the inflammatory and nutritional factors screened by LASSO regression and the baseline clinical variable were analyzed by univariate analysis and multivariate analysis to determine the independent predictive factors. *P* value < 0.10 in the univariate analysis was put into the multivariate analysis. Fourth, we established one novel nomogram model based on the determined independent predictive factors. We evaluated the nomogram prediction ability through ROC and area under the curve (AUC). The consistency between the predicted results and the actual results was measured with the correction curve, and the clinical value of the Nomogram model was further evaluated with the decision curve analysis (DCA). A total of 30 cases were randomly selected to internally validate the nomogram model using ROC, calibration curve, and DCA curve. We use R software (version 3.6.3) and Python (version 3.7) for statistical analysis. Bilateral *P* value <0.05 is statistically significant in this study.

## Results

### Comparisons of baseline characteristics between the pCR group and the non-pCR group

A total of 97 patients were included for further analysis, with 20 (20.62%) patients in the pCR group and 77 (79.38%) patients in the non-pCR group. The clinical and demographic characteristics of the two groups were comparable, including sex, age, left ventricular ejection fraction (LVEF), American Society of Anesthesiologists (ASA) status, drinking history, smoking history, diabetes, hypertension, tumor location, forced expiratory volume in one second (FEV1), and tumor location (*P* > 0.05). The non-pCR group had a higher clinical stage, but the difference wasn't significant (*P* = 0.06). The neoadjuvant chemotherapy regiment, neoadjuvant cycle, and PD-1 drug type were similar in both groups. The time to surgery was 42 days and 41 days in the pCR group and non-pCR group, respectively. The comparisons of baseline characteristics are summarized in Table 1.

**Table 1:** Comparisons of baseline characteristic between the pCR group and non-pCR group.

Contents	Total	pCR group (*n* = 77)	non-pCR group (n = 20)	*p*
sex, *n* (%)				0.88
Female	23 (23.71)	18 (23.38)	5 (25.00)	
Male	74 (76.29)	59 (76.62)	15 (75.00)	
Age, mean (±SD)	60.35 ± 6.73	60.44 ± 6.38	60.00 ± 7.94	0.80
LVEF, mean (±SD)	67.21 ± 5.47	67.24 ± 5.83	67.09 ± 3.75	0.90
FEV1, mean (±SD)	2.58 ± 0.63	2.56 ± 0.62	2.69 ± 0.65	0.42
ASA status, *n* (%)				0.67
2	90 (92.78)	71 (92.21)	19 (95.00)	
3	7 (7.22)	6 (7.79)	1 (5.00)	
Smoking History, *n* (%)				0.86
No	42 (43.30)	33 (42.86)	9 (45.00)	
Yes	55 (56.70)	44 (57.14)	11 (55.00)	
Dringking History, *n* (%)				0.75
No	65 (67.01)	51 (66.23)	14 (70.00)	
Yes	32 (32.99)	26 (33.77)	6 (30.00)	
Hypertension, *n* (%)				0.41
No	79 (81.44)	64 (83.12)	15 (75.00)	
Yes	18 (18.56)	13 (16.88)	5 (25.00)	
Diabetes, *n* (%)				0.81
No	91 (93.81)	72 (93.51)	19 (95.00)	
Yes	6 (6.19)	5 (6.49)	1 (5.00)	
Tumorlocation, *n* (%)				0.87
Upper third	9 (9.28)	7 (9.09)	2 (10.00)	
Middle third	49 (50.52)	38 (49.35)	11 (55.00)	
Lower third	39 (40.21)	32 (41.56)	7 (35.00)	
Clinical stage, *n* (%)				0.06
≦2	40 (41.24)	28 (36.36)	12 (60.00)	
>2	57 (58.76)	49 (63.64)	8 (40.00)	
Drugs type, *n* (%)				0.98
Pembrolizumab	39 (40.21)	31 (40.26)	8 (40.00)	
Others	58 (59.79)	46 (59.74)	12 (60.00)	
Neoadjuvant cycles, *n* (%)				0.66
≦2	67 (69.07)	54 (70.13)	13 (65.00)	
>2	30 (30.93)	23 (29.87)	7 (35.00)	
Chemotherapy regimens, *n* (%)				0.38
TP regiment	87 (89.69)	68 (88.31)	19 (95.00)	
PF regiment	10 (10.31)	9 (11.69)	1 (5.00)	
Time to surgery, median[IQR]	41[33,50]	42[33,52]	41[34,44]	0.38

ASA, American Society of Anesthesiologists; FEV1, forced expiratory volume in one second; LVEF, left ventricular ejection fractions.

**Figure 1:**
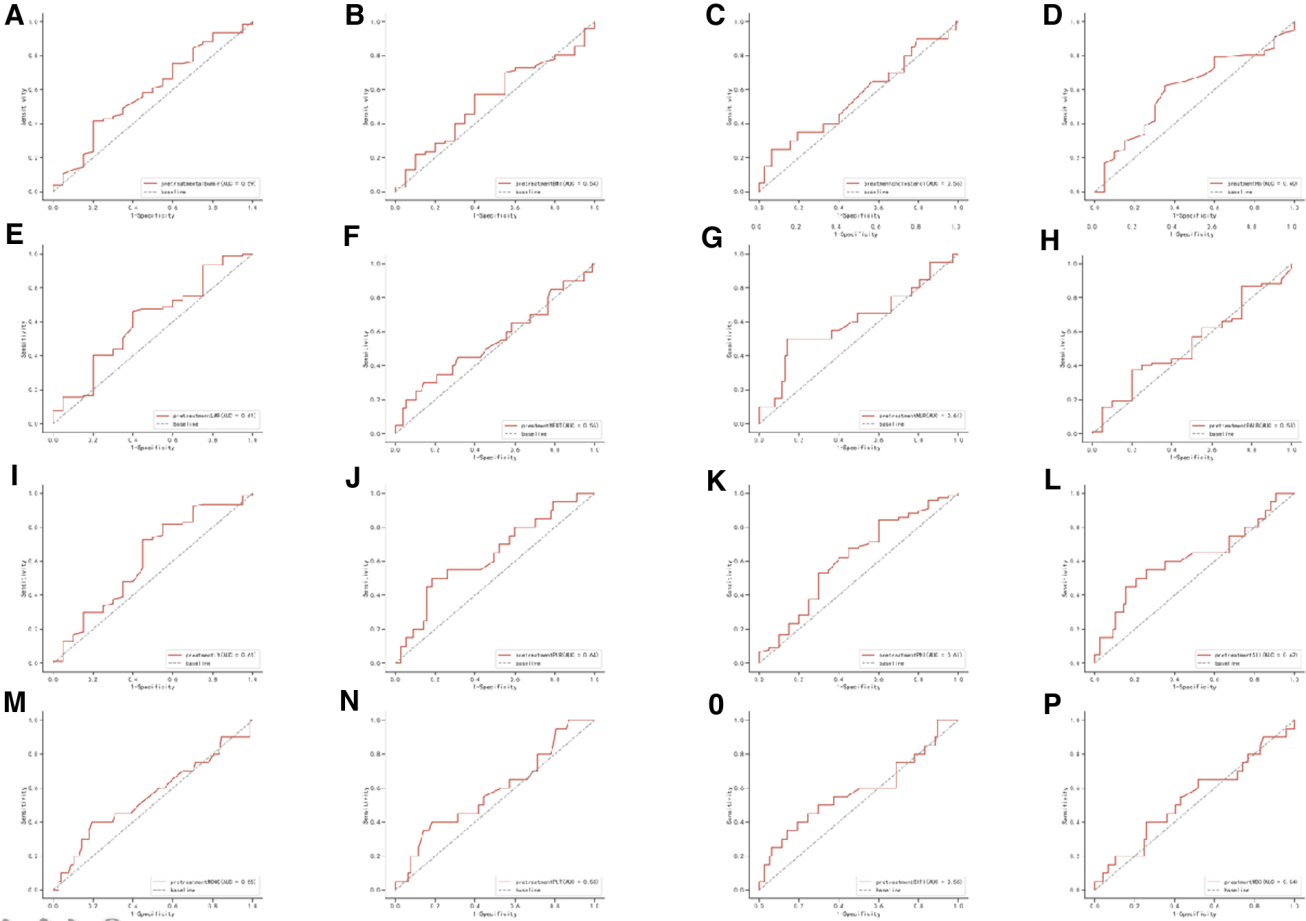
Receiver operating characteristic (ROC) of the following 16 predictive factors: HALP, SIRI, SII, PNI, PLR, LMR, NLR, BMI, PLT, Hb, MONO, LY, NEUT, WBC, cholesterol, and Alb.

The details of comparisons of inflammatory and nutritional parameters between the pCR group and the non-pCR group were summarized in Table 2. Compared with the non-pCR group, the pCR group had a higher SII (median 871.72 vs. 614.71), but not significant. In addition, we summarized the ROC of the included inflammatory and nutritional parameters in Figure 1.

**Table 2:** Comparisons of preteratment inflammatory and nutritional indicators between the pCR group and non-pCR group.

Contents	Total	Non-pCR group (*n* = 77)	pCR group (*n* = 20)	*p*
Pretreatment HALP, median[IQR]	43.00[19.00,67.00]	43.00[19.00,68.00]	48.00[25.00,55.00]	0.66
Pretreatment SIRI, median[IQR]	0.98[0.65,1.41]	0.98[0.65,1.32]	1.19[0.69,1.98]	0.27
Pretreatment SII, median[IQR]	635.31[414.18,878.57]	614.71[414.18,844.79]	871.72[474.24,1127.46]	0.11
Pretreatment PNI, median[IQR]	50.30[47.25,54.15]	51.00[47.80,54.25]	49.10[45.60,52.30]	0.12
Pretreatment PLR, median[IQR]	152.35[111.96,200.69]	147.87[108.16,184.67]	201.52[140.31,210.35]	0.06
Pretreatment LMR, median[IQR]	4.25[3.46,5.61]	4.31[3.52,5.62]	3.96[3.46,4.90]	0.15
Pretreatment NLR, median[IQR]	2.45[1.79,3.35]	2.42[1.79,3.04]	3.59[1.98,3.90]	0.14
Pretreatment BMI, median[IQR]	20.83[19.53,22.43]	20.94[19.53,22.72]	20.58[19.53,22.21]	0.55
Preatment weight, median[IQR]	57.00[53.00,62.00]	57.00[53.00,62.00]	57.00[54.00,60.50]	0.95
Pretreatment PLT, mean (±SD)	257.90 ± 56.21	254.30 ± 55.81	271.75 ± 55.61	0.22
Preatment Hb, mean (±SD)	139.13 ± 15.21	139.81 ± 15.48	136.55 ± 13.82	0.40
Pretreatment MONO, median[IQR]	0.40[0.33,0.48]	0.40[0.33,0.48]	0.41[0.34,0.55]	0.46
Preatment LY, median[IQR]	1.70[1.37,2.08]	1.70[1.45,2.13]	1.48[1.22,1.96]	0.13
Preatment NEUT, median[IQR]	4.17[3.40,4.95]	4.17[3.41,4.82]	4.22[3.38,5.84]	0.50
Preatment WBC, median[IQR]	6.52[5.60,7.77]	6.52[5.60,7.74]	6.92[5.76,7.77]	0.62
Preatment cholesterol, median[IQR]	4.74[4.25,5.30]	4.74[4.23,5.20]	4.74[4.35,5.52]	0.41
Pretreatment albumin, median[IQR]	41.70[38.80,44.20]	41.70[39.10,44.30]	41.20[38.10,42.90]	0.23

### Screening predictive inflammatory nutritional indicators using LASSO-logistic regression analysis

**Figure 2:**
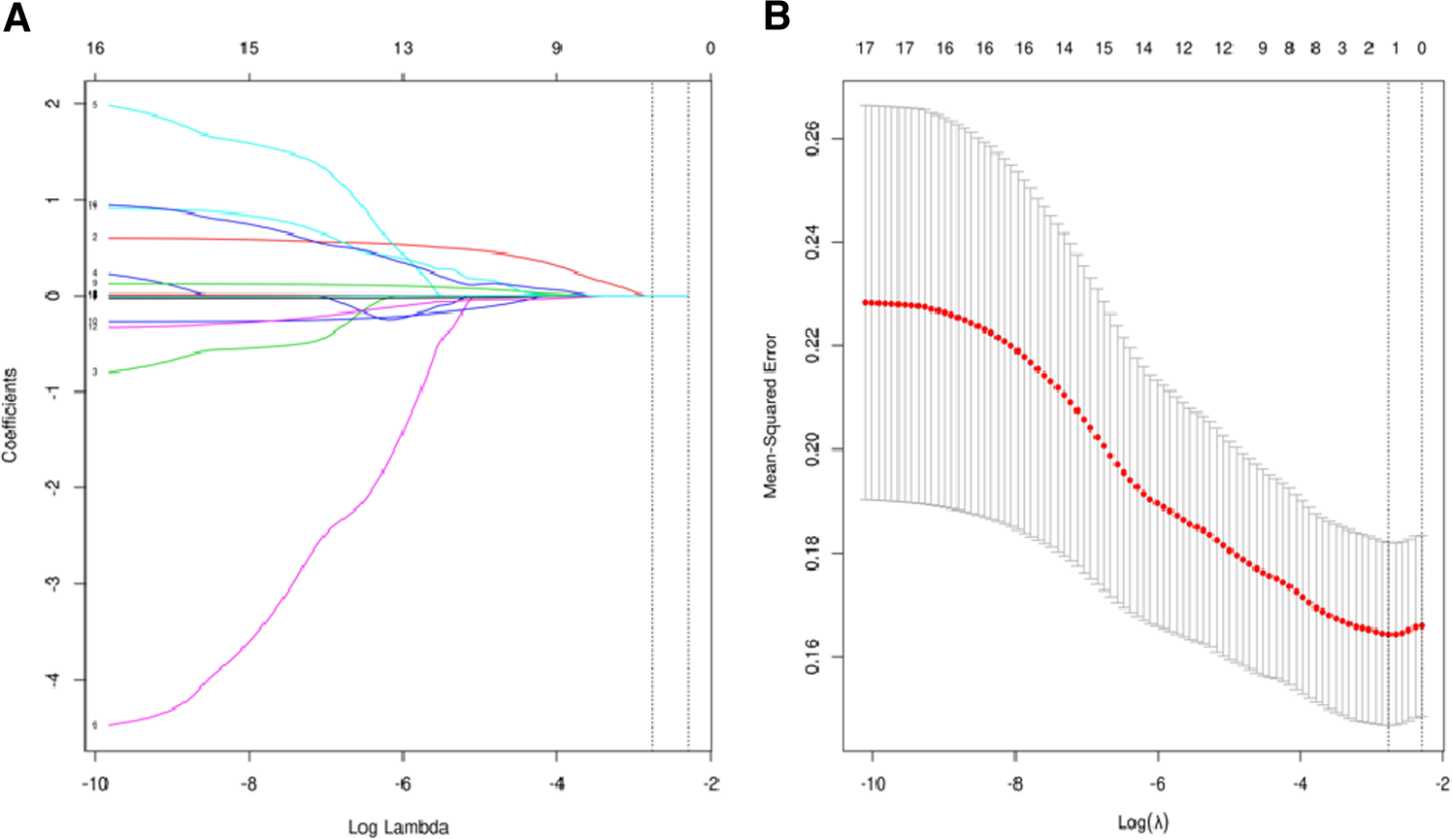
(**A**) regression analysis of influence factors based on Lasso for variable selection; (**B**) cross-validation of the regression model.

Considering the potential collinearity between inflammatory nutritional indicators, we used LASSO regression analysis (Figure 2A) and cross-validation (Figure 2B) for each predictive factor to screen the independent variables. A total of 17 potential factors were put into the LASSO analysis, including HALP, SIRI, SII, PNI, PLR, LMR, NLR, BMI, weight, PLT, Hb, MONO, LY, NEUT, WBC, cholesterol, and Alb. The smallest verification error (*λ*)was 0.06, and only one predictive factor (SII) was included in the regression model. The cut-off value of SII was 921.80, with an AUC value of 0.62. Further, we defined SII > 921.80 as high SII and SII ≦ 921.80 as low SII.

### Univariate analysis and multivariate analysis of pCR predictive factors

To determine the independent factors of pCR, we conducted univariate and multivariate analyses to determine the independent predictive factors, and finally, two factors were screened. High SII (OR = 3.94, 95%CI:1.26–12.42, *P* = 0.02) and early clinical stage(OR = 0.35, 95%CI:0.12–0.98, *P* = 0.05) were determined as independent predictive factors of pCR. The analysis details are summarized in Table 3.

**Table 3:** Univariate analysis and multivariate analysis of pCR predictive factors.

Variables	N	OR	95%CI	*P*	OR	95%CI	*P*
Sex							
Female	23						
Male	74	0.92	[0.29,2.87]	0.88			
Age							
≦51	10						
>51	87	0.57	[0.13,2.42]	0.44			
PD-1 type, *n* (%)							
Pembrolizumab	58						
Others	39	0.99	[0.36,2.70]	0.98			
Neoadjuvant cycles, *n* (%)							
≦2	67						
>2	30	1.26	[0.45,3.58]	0.66			
Chemotherapy regiment							
TP regiment	87						
PF regiment	10	0.40	[0.047,3.34]	0.40			
Time to surgery							
≦45	65						
>45	32	0.44	[0.133,1.44]	0.17			
Pretreatment SII							
≦921.80	77						
>921.80	20	3.61	[1.22,10.70]	0.02	3.94	[1.26,12.42]	0.02
ASAstatus							
2	90						
3	7	0.62	[0.07,5.49]	0.67			
Smokinghistory							
No	42						
Yes	55	0.92	[0.34,2.47]	0.86			
Dringkinghistory							
No	65						
Yes	32	0.84	[0.29,2.44]	0.75			
Hypertension							
No	79						
Yes	18	1.64	[0.51,5.31]	0.41			
Diabetes							
No	91						
Yes	6	0.76	[0.08,6.88]	0.81			
FEV1202							
≦2.02	24						
>2.02	73	2.13	[0.56,8.00]	0.27			
LVEF							
≦70.5	70						
>70.5	27	0.39	[0.10,1.46]	0.16			
Tumorlocation							
Upper	9						
Middle third	49	1.01	[0.18,5.60]	0.99			
Lower third	39	0.77	[0.13,4.50]	0.77			
Clinical stage							
≦2	40						
>2	57	0.38	[0.14,1.04]	0.06	0.35	[0.12,0.98]	0.05

### Establishment and validation of the nomogram model

**Figure 3:**
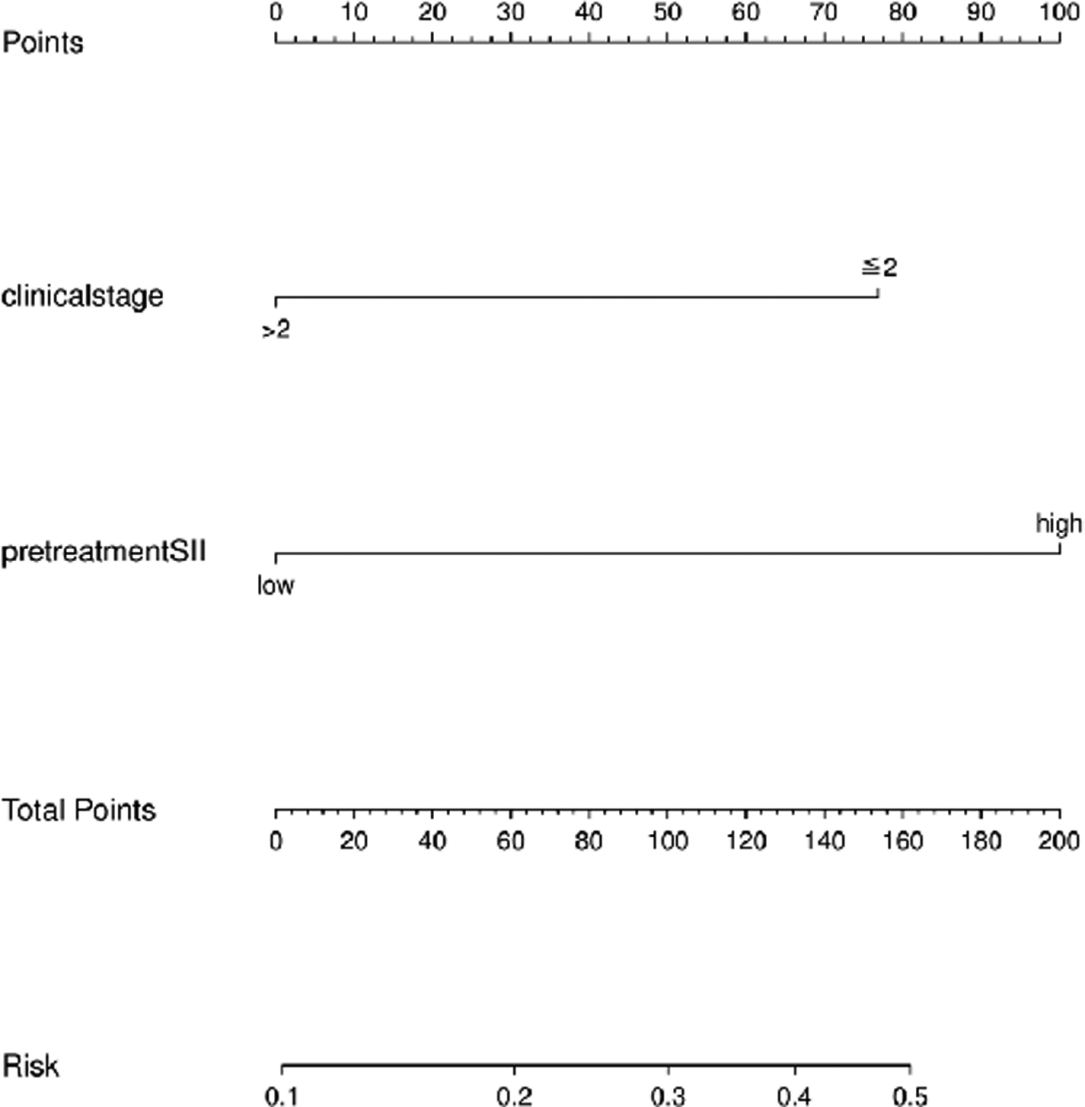
Nomogram model to predict pCR among ESCC patients receiving nICT.

**Figure 4:**
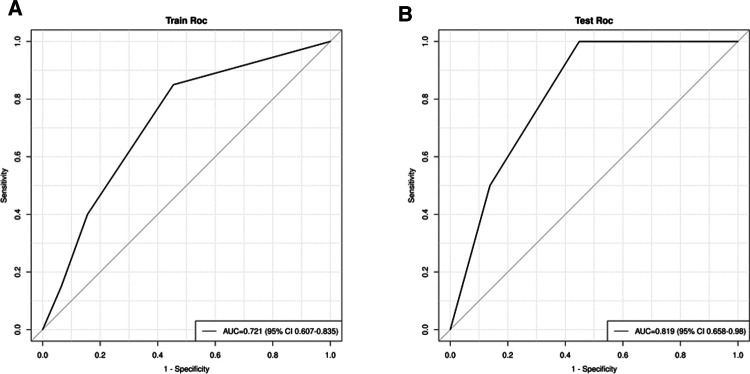
(**A**) receiver operating characteristic (ROC) in the training cohort; (**B**) ROC in the validation cohort.

**Figure 5:**
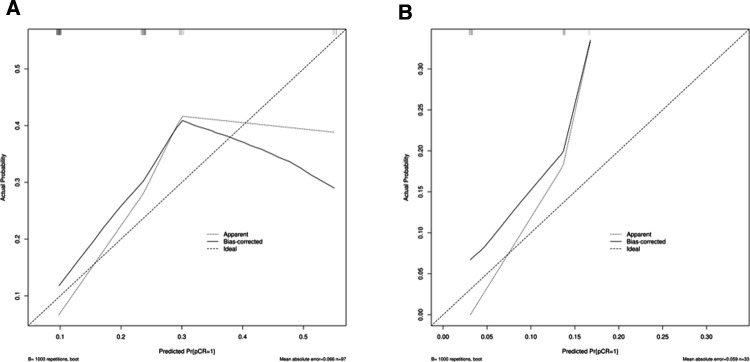
(**A**) Calibration curve in the training cohort; (**B**) calibration curve in the validation cohort.

**Figure 6:**
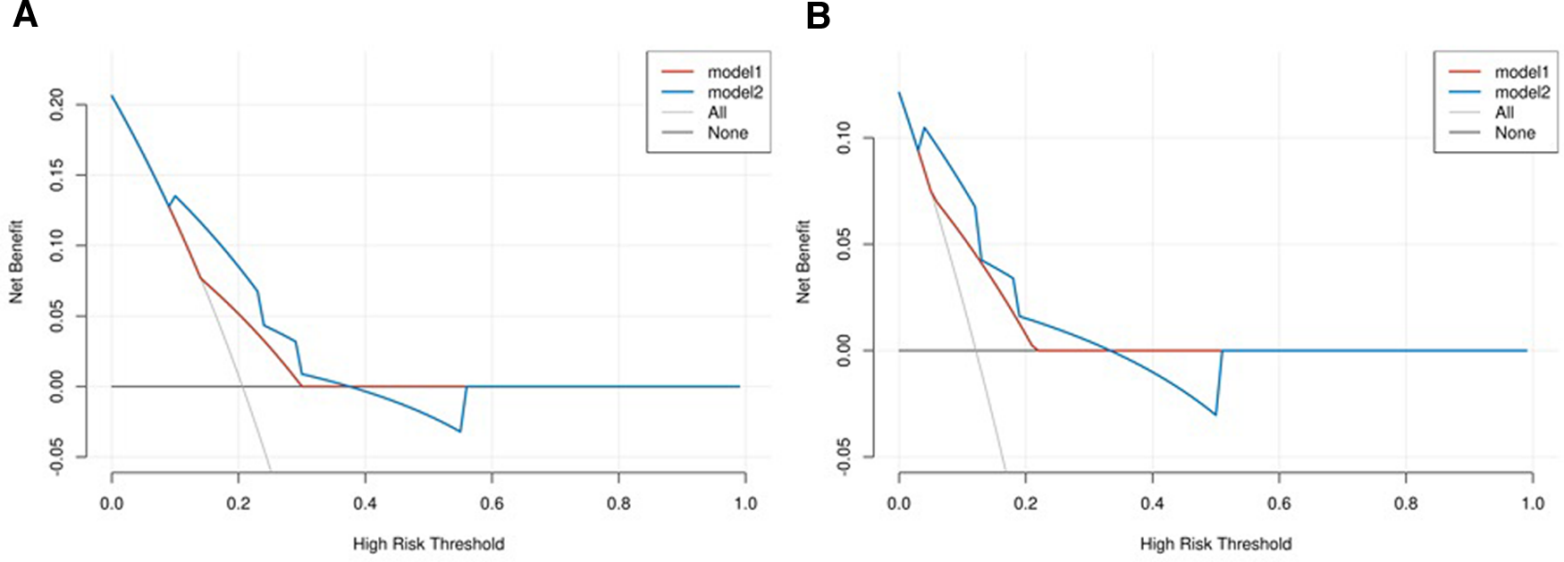
(**A**) Decision curve analysis (DCA) in the training cohort; (**B**) DCA in the validation cohort. The model 1 stands for clinical stage, and the model 2 stands for combination of clinical stage and SII.

We combined clinical stage and SII to establish a novel nomogram model to predict the pCR(Figure 3). The established nomogram model showed good discriminative ability in both the training cohort and validation cohort, with an AUC 0.72 (95% CI:0.61–0.84) and 0.82(95%CI: 0.66–0.98) (Figure 4). The Brier score of the calibration curve was 0.13, which was below 0.25. Thus, the calibration curve showed good agreement between the predicted results and the actual results in both the training cohort and the validation cohort (Figure 5). Compared with the clinical stage, the DCA confirmed a better clinical value of the nomogram model in both the training cohort and the validation cohort (Figure 6).

## Discussion

The pCR is an important evaluation index of the short-term efficacy of neoadjuvant therapy for ESCC, which was closely also associated with improved long-term overall survival and decreased recurrence. The JCOG9907 trial showed that the pCR rate among EC patients receiving neoadjuvant chemotherapy was only 2.4% (19). In this study, a total of 20 (20.62%) patients achieved pCR. Recently, a meta-analysis included 621 resectable esophageal cancer patients receiving neoadjuvant immunotherapy, and among them, 33.8% (95% CI: 29.6%-37.9%) patients achieved pCR (20). Based on present evidence, the efficacy of the nICT pattern is promising and has the potential to be the standard treatment of locally advanced esophageal cancer. Thus, the prediction of independent predictive factors for pCR and the establishment of accurate prediction models are of great importance for the formulation of individual neoadjuvant therapy. In this study, we identified early clinical stage and high SII as independent predictors of pCR and established a novel Normogram model for predicting pCR among patients receiving nICT. The model had a good discriminant ability, with an AUC of 0.72 in the training queue and 0.82 in the verification queue. Using the nomogram model, each predictive factor was quantified and visualized by the model to predict the probability of pCR. In addition, physicians could predict an individual's response to nICT and personalize neoadjuvant treatment plans.

The SII uses a simple calculation based on lymphocyte, neutrophil, and platelet counts in peripheral blood to evaluate patients' immune status objectively and is widely reported as a prognostic marker of multiple malignant tumors. Chen et al. retrospectively analyzed 1,383 patients undergoing radical surgery for colorectal cancer and found low SII was associated with longer overall survival and disease-free survival (21). Wang et al. found that high SII could be used as an independent predictor of poor prognosis in patients with stage I-III gastric cancer and was superior to NLR and PLR (22). Feng JF et al. confirmed that ESCC patients with SII ≤ 410 had a significantly better 5-year cancer-specific survival (51.9% vs. 24.0%) (23). The predictive value of SII among ESCC patients receiving nICT was rarely reported.

In this study, we found that high SII(OR = 3.94, 95%CI:1.26–12.42, *P* = 0.02) was associated with better treatment response. Recently, Xinke Z et al. combined NLR, LMR, PLR, and SII to predict the pathological effect of anti-PD-1 combined with neoadjuvant chemotherapy in ESCCpatients (24). In Xinke Z's analysis, patients with treatment response had high baseline SII, and the cut-off value of SII at baseline was 559.266 with an AUC value of 0.681 (14), which also indicated a positive correlation between the SII and pathological response. The PD-1 blockade is designed to inhibit the interaction between PD-1 and PD-L1to activate T cells, which helps in restoring the anti-cancer immune response. Despite the promising results of PD-1, drug resistance is considered a major problem in PD-1 treatment because a large proportion of patients couldn't respond to PD-1 at the beginning of treatment (25). Tumor-associated neutrophils have been reported to indirectly promote the antitumor function of CD8 + T cells by regulating interleukin (IL)-17 production (26). However, the mechanisms of high SII associated with a better treatment response among ESCC patients receiving nICT were unclear. At present, we are conducting single-cell sequencing analysis to examine the difference in cell distribution in patients with response to nICT and patients without response to nICT, and the study is still in the data collection stage. We would put SII as a subgroup factor in the following analysis and hope to give a clear explanation of this finding.

To our best knowledge, this study first investigated the predictive value of SII in the prediction of pCR and established one nomogram model to predict pCR among patients receiving nICT. However, this study has the following limitations: First, the analysis lacks data randomization, and the study may have a potential bias in patient selection and processing of missing values. Second, although the prediction model has good discriminative power, however, it only includes relatively limited cases whose pathological type is squamous cell carcinoma, and it has not been verified externally. Therefore, further external validation is necessary before applying the Nomogram model to patients in other centers. Third, the impact of SII on the long-term Four, it is unclear whether this nomogram will be suitable for patients with locally advanced esophageal cancer receiving other neoadjuvant therapy, such as neoadjuvant chemotherapy and neoadjuvant chemoradiotherapy. Fifth, the mechanism should be further investigated using single-cell sequencing analysis.

## Conclusions

High pretreatment SII and early clinical stage were independently associated with pCR among ESCC receiving nICT. Calculation of SII is based on routine preoperative hematologic indicators. We further established and validated one nomogram model to predict pCR among ESCC receiving nICT, which is easy to be applied in clinical decision-making, and the evaluation process is simple and feasible. Considering the relatively limited case number from a single center, external validation, including more cases, are necessary to support our findings.

## Data Availability

The original contributions presented in the study are included in the article/Supplementary Material, further inquiries can be directed to the corresponding author/s.
